# The effects of probiotics on total cholesterol: A meta-analysis of randomized controlled trials

**DOI:** 10.1097/MD.0000000000009679

**Published:** 2018-02-02

**Authors:** Lang Wang, Mao-Juan Guo, Qing Gao, Jin-Feng Yang, Lin Yang, Xiao-Li Pang, Xi-Juan Jiang

**Affiliations:** a College of Nursing; b School of Integrative Medicine, Tianjin University of Traditional Chinese Medicine; c Hospital of Ren Min, JiXian, Tianjin, China.

**Keywords:** a meta-analysis, probiotics, randomized controlled trials, total cholesterol

## Abstract

**Background:**

Probiotics supplements provide a new nonpharmacological alternative to reduce cardiovascular risk factors. The impact of probiotics on the reduction of total cholesterol (TC) remains controversial. We conducted a meta-analysis to showcase the most updated and comprehensive evaluation of the studies.

**Methods:**

Randomized controlled trials (RCTs) were searched from electronic databases, including PubMed, Embase, Cochrane Central Register of Controlled Trials, Chinese Biomedical Literature Database, China National Knowledge Infrastructure, Wanfang database dating from January 2007 to January 2017. The curative effects of probiotics on the reduction of TC were assessed using mean difference (MD), as well as their 95% confidence interval (CI). RevMan software (version 5.3) was used to carry out this meta-analysis.

**Results:**

Thirty-two RCTs including 1971 patients met the inclusion criteria. Results of this analysis showed that compared with the control group serum TC was significantly reduced in probiotics group [MD = −13.27, 95% CI (−16.74 to 9.80), *P* < .05]. In addition, specific strains also significantly reduced serum TC, *L acidophilus* and *B lactis* [MD = −8.30, 95% CI (−10.44, −6.15), *P* < .05]; VSL#3 [MD = −11.04, 95% CI (−19.61, −2.48), *P* < .05]; *L plantarum t* ≤ 6 weeks: [MD = −1.56, 95% CI (−6.97, −3.86), *P* < .05] or *t* > 6 weeks: [MD = −22.18, 95% CI (−28.73, −15.63), *P* < .05]. Subgroup analysis indicated that the difference of baseline TC, probiotics forms and intervention duration might have a significant impact on the results. However, strains and doses of probiotics had no significant influence on curative effects.

**Conclusion:**

Available evidence indicates that probiotics supplements can significantly reduce serum TC. Furthermore, higher baseline TC, longer intervention time, and probiotics in capsules form might contribute to a better curative effect.

## 1 Introduction

Cardiovascular disease poses a serious threat to human life, and 17.9 million individuals died from cardiovascular disease in 2015, which rose by 12.5% since 2005.^[1]^ Epidemiological studies have confirmed the correlation between total cholesterol (TC) with increased cardiovascular risk.^[2]^ Apart from pharmaceutical approaches, probiotics therapy also showed a potential effect in TC regulation. Therefore, probiotics triggered a great interest among researchers to treat cardiovascular disease. Probiotics are defined as living microorganisms that confer a health benefit, when they are administered in adequate amounts,^[3,4]^ and it is widely used nowadays.

A large number of in vitro and in vivo studies have shown that probiotics do have hypolipidemic effects.^[5–8]^ However, the lipid lowering effect of probiotics is controversial in human clinical studies. Some researches argue against this role,^[9,10]^ while 6 earlier meta-analyses approved this hypolipidemic role.^[11–16]^ These 6 studies, however, suffered from some flaws, such as limitation of quantity and quality of literatures,^[11,13–16]^ single database,^[12]^ only including English literatures,^[11–16]^ short intervention time,^[11,16]^ missing subgroup analysis,^[11,13,15]^ nonreporting of publication bias.^[11–16]^

Probiotics supplements provide a novel nonpharmacological alternative to reduce cardiovascular risk factors. In order to investigate the effect of probiotics on serum TC under different conditions, we conducted a meta-analysis to show the most updated and comprehensive summary of previous randomized controlled trials (RCTs). Our aim is to explore the effects of different probiotics on serum TC. In addition, we assess whether these effects influenced by factors such as study design, baseline TC level, or strains, doses, forms, and intervention duration of probiotics.

## 2 Methods

### 2.1 Search strategy

Electronic databases include PubMed, Embase, Cochrane Central Register of Controlled Trials (CENTRAL), Chinese Biomedical Literature Database, China National Knowledge Infrastructure, and Chinese Wanfang database were used for literature mining. Articles published in both English and Chinese from January 2007 to January 2017 were searched against. The following keywords were used: (Probiotics OR Streptococcus OR Bifidobacterium OR Enterococcus OR Lactococcus OR Lactobacillus OR Bacillus OR Saccharomyces OR yogurt OR yoghurt OR “fermented milk” OR “sour milk”) AND (Cholesterol OR TC OR “lipid profile” OR “plasma lipids” OR “serum lipids” OR “Lipids/blood” OR HDL-cholesterol OR “Cholesterol, HDL” OR LDL-cholesterol OR “Cholesterol, LDL” OR TGs OR Triglycerides) AND (random∗ OR “Randomized Controlled Trials as Topic” OR “Randomized Controlled Trial[Publication Type]”).

### 2.2 Inclusion and exclusion criteria

The inclusion criteria in our meta-analysis were: The study design was randomized controlled trial; enrolling adult patients (above 18 years old) that without any other medical conditions; probiotics products used in study should contain living bacteria; studies which include means and standard deviations of TC. On the other side, exclusion criteria were as follows: probiotics products containing prebiotics or plant sterols; subjects who had previously undergone intestinal surgery; pregnant women; duplication of previous research; articles without full text access.

### 2.3 Data extraction and quality assessment

The studies were selected by 2 independent researchers based on inclusion and exclusion criteria. Any disagreement between researchers was resolved by consulting the third researcher. Then following information was collected: first author's name, publication time, study location, health status and age of participants, baseline TC, group information, study design, intervention duration, sample size, additionally, strain, dose, form of probiotics (Table 1).

**Table 1:**
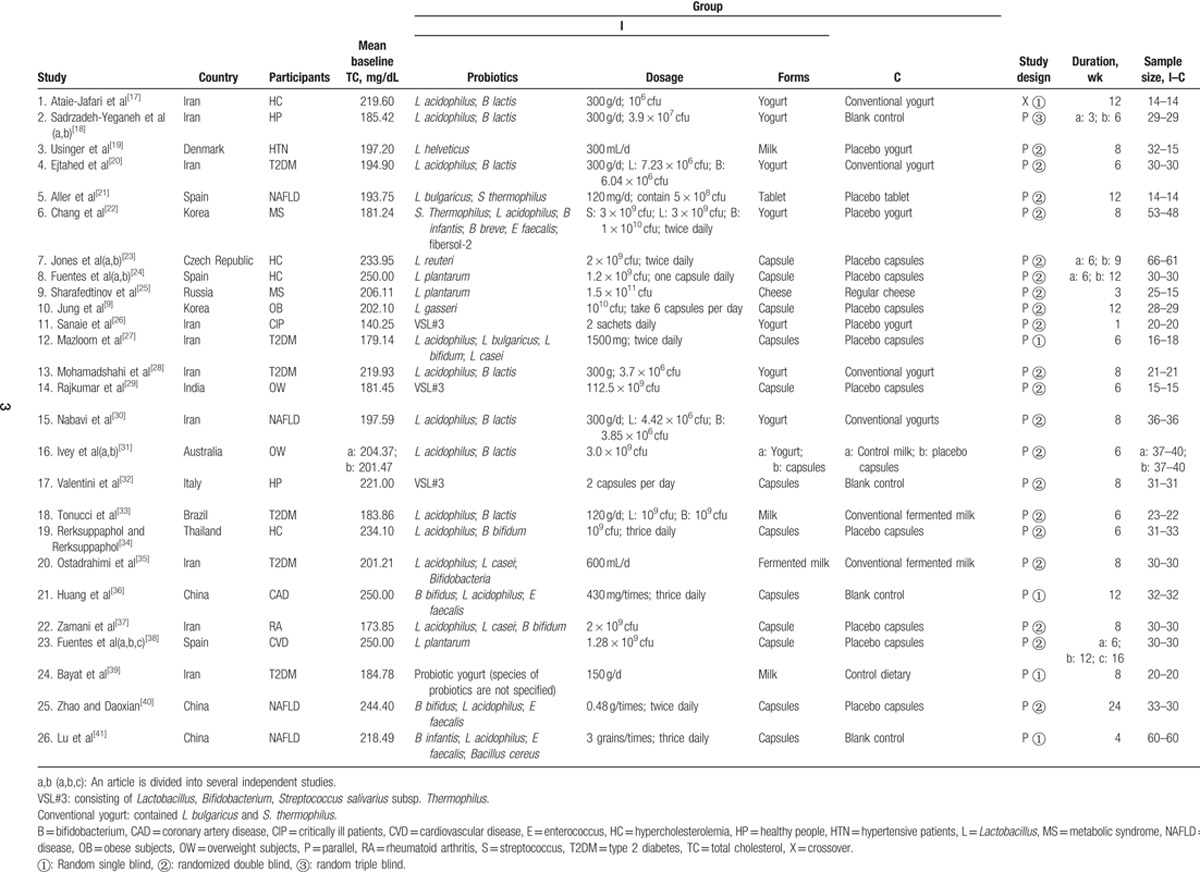
Characteristics of included studies.

The quality of the studies was independently evaluated by 2 researchers according to the Cochrane Handbook for Systematic Reviews of Interventions, version 5.1.0.

### 2.4 Data analysis

Before meta-analysis, the lipid levels in mmol/L were all converted to mg/dL. The conversion factor was 1 mmol/L = 38.67 mg/dL for cholesterol. The mean net changes (mean values ± standard deviation) of TC level after probiotics treatment in each study were calculated. For parallel trials, mean net change was calculated as endpoint subtract by baseline; for crossover trials, mean net change was the difference in TC concentrations between the intervention and controlled periods. And, standard errors were converted to standard deviation for the analyses. The homogeneity among trials was evaluated by *P* (or *I*^2^). If *P* ≥ .1 (*I*^2^ ≤ 50%), the studies were considered to be homogeneous, then a fixed-effects model was used. If *P* < .1 (*I*^2^ > 50%), the trials were considered to be heterogeneous, then a random effects model was used. Summary statistics was calculated using 2-sided *P*-value as criteria to determine the statistically significance. *P* < .05 was denoted as statistically significant. In order to explore the influence of other factors on the results, a series of subgroup analyses were carried out. These factors include baseline TC, strain, dose, form, and intervention duration of probiotics. Furthermore, meta-regression was conducted to investigate sources of heterogeneity. A sensitivity analysis was conducted to investigate the stability of the combined results. And the funnel plot was used to investigate the existence of publication bias; we also calculated the fail-safe number. Formula: Nfs0.05 = (*Z*/1.64) 2-*K*. *Z* is the *Z*-value of each independent study, *K* is the number of included studies, taking *P* = .05. All analyses were performed using Review Manager 5.3 (Cochrane Collaboration, Copenhagen, Denmark) and Stata 14.0 (StataCorp, College Station, TX).

## 3 Results

### 3.1 Selection of trials

A total of 1438 articles were retrieved, including 1012 English articles and 426 Chinese articles. Duplicate articles (n = 327) were removed then another 995 articles were removed by reading their titles and abstracts. Finally, 24 articles were selected after full text reading, and 2 additional articles^[9,21]^ were included from other sources. Twenty-six articles that met the inclusion criteria were identified. There into, 5 articles can be viewed as multiple studies. There are studies that applied more than one intervention times so can be regarded as multiple independent studies, which include Sadrzadeh-Yeganeh et al's^[18]^ study (2), Jones et al's study^[23]^ (2), Fuentes et al's study^[24]^ (2), Fuentes et al's study^[38]^ (3). Another study used 2 different delivery methods of milk or yogurt by Ivey et al,^[31]^ which can also be considered as 2 independent studies. Thus, a total of 32 independent studies were included in the analysis. The characteristics of these including articles are shown in Table1.

### 3.2 Quality assessment

We evaluated the quality of the literatures, mainly in 7 aspects: random sequence generation, allocation concealment, blinding of participants and personnel, blinding of outcome assessment, incomplete outcome data, selective outcome reporting, and “other issues.” All studies were randomized, but 10 studies did not clearly stated the random sequence generation methods.^[9,17–19,24,26,29,36,38,39]^ One study was assessed as high risk in allocation concealment,^[17]^ 15 as unclear risk,^[21,22,24,25,27,29,30,32,37,40]^ and others 10 studies as low risk of bias. One study did not use the blind of participants and personnel,^[27]^ and 3 articles were not clear.^[17,39,41]^ Three studies provided incomplete outcome data.^[22,28,34]^ All studies carried out a complete outcome reporting. And almost all the trials did not give a clear description of blinding of outcome assessment and other issues. Although some projects were assessed as high risk, the overall literature quality is at a moderate level. Therefore, the included literatures can be subjected to meta-analysis.

### 3.3 The effect of probiotics on TC

**Figure 1:**
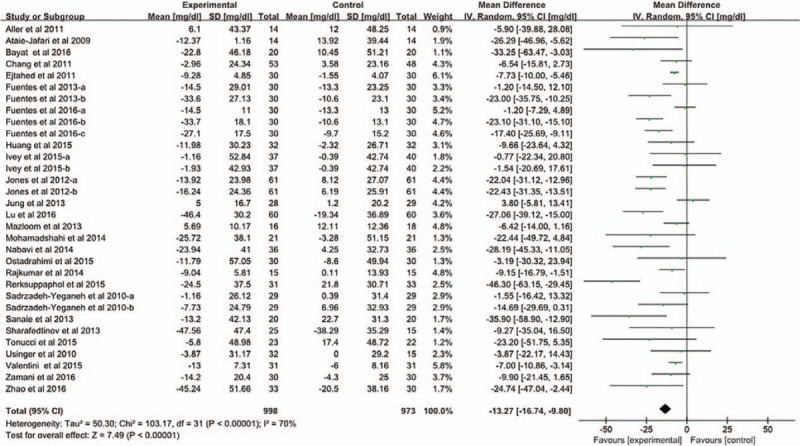
The effect of probiotics on TC. We conducted a meta-analysis of 32 studies. And, we evaluated homogeneity by *Q* test. The pooled mean difference in TC was [MD = −13.27, 95% CI (−16.74–9.80), *P* < .05] in the random-effect model analysis.

The total 32 studies enrolling 1971 participants that were used to describe the changes (I–C) of TC for meta-analysis. The results showed with obvious heterogeneity among the studies (*P* < .1, *I*^2^ = 70%). The pooled mean net change in TC for those treated with Probiotics compared to controls was [mean difference—MD = −13.27, 95% confidence interval—CI (16.74–9.80), *P* < .05] in the random-effect model analysis (Fig. 1). Sensitivity analysis was conducted. As a result, heterogeneity persists. In order to account for the heterogeneity, different models were used, and the pooled mean net change in TC was [MD = −9.75, 95% CI (−11.21, −8.29), *P* < .05] in the fixed-effects model analysis. The results of the 2 models are similar. So the meta-analysis results can be considered reliable.

### 3.4 The effect of specific strains on TC (inclusion criteria: The studies ≥ 3)

**Figure 2:**
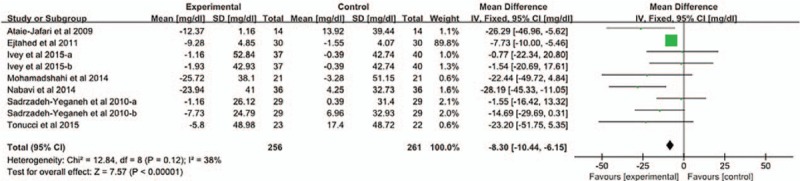
The effect of 2 strains of probiotics (*L acidophilus*; *B lactis*) on TC. The 9 study selected probiotics for *L acidophilus* and *B lactis*. We conducted a meta-analysis to investigate the effects of these 2 strains on TC. The pooled mean difference in TC was [MD = −8.30, 95% CI (−10.44, −6.15), *P* < .05] in the fixed-effects model analysis.

Nine studies used strains of *L acidophilus* and *B lactis* is for intervention. Meta-analysis was conducted to investigate the effects of these 2 strains on TC. Heterogeneity was not detected (*P* = .12, *I*^2^ = 38%). The pooled mean net change in TC was [MD = −8.30, 95% CI (−10.44, −6.15), *P* < .05] in the fixed-effects model analysis (Fig. 2).

**Figure 3:**
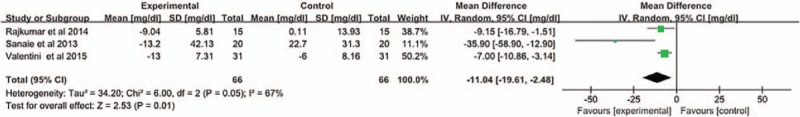
The effect of VSL#3 on TC. The 3 study selected probiotics for VSL#3. We conducted a meta-analysis to investigate the effects of VSL#3 on TC. The pooled mean difference in TC was [MD = −11.04, 95% CI (−19.61, −2.48), *P* < .05] in the random-effects model analysis.

Three studies selected probiotics of VSL#3. The result showed that heterogeneity existed among studies (*P* = .05, *I*^2^ = 67%). The pooled mean net change in TC was [MD = −11.04, 95% CI (−19.61, −2.48), *P* < .05] in the random-effect model analysis (Fig. 3).

**Figure 4:**
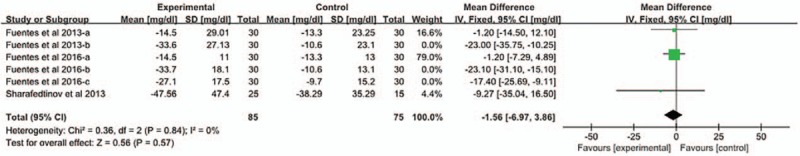
The effect of *L plantarum* on TC (short intervention cycle).

**Figure 5:**
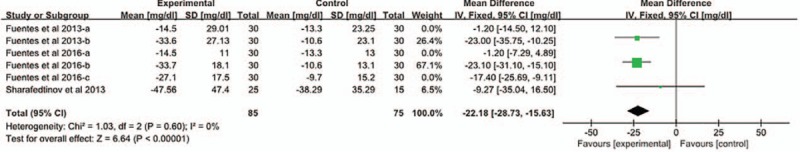
The effect of *L plantarum* on TC (long intervention cycle). The 6 study selected probiotics for *L plantarum*. The analysis showed that the heterogeneity was large (*P* < .1, *I*^2^ = 81%). We divided the study into 2 subgroups according to the time of intervention. Figure 4 shows the results of the short intervention cycle. The pooled mean difference in TC was [MD = −1.56, 95% CI (−6.97, −3.86), *P* < .05] in the fixed-effects model analysis; this figure shows the results of the long intervention cycle. The pooled mean difference in TC was [MD = −22.18, 95% CI (−28.73, −15.63), *P* < .05] in the fixed-effects model analysis.

Six studies used strains of *L plantarum* for intervention. The analysis showed that large heterogeneity existed among studies (*P* < .1, *I*^2^ = 81%). The studies were divided into 2 subgroups according to the time of intervention. Figure 4 shows the results of the short intervention cycle (*t* ≤ 6 weeks). No heterogeneity (*P* = .84, *I*^2^ = 0%). The pooled mean net change in TC was [MD = −1.56, 95% CI (−6.97, −3.86), *P* < .05] in the fixed-effects model analysis; Figure 5 describes the results of the long intervention cycle (*t* > 6 weeks). No heterogeneity (*P* = .60, *I*^2^ = 0%). The pooled mean net change in TC was [MD = −22.18, 95% CI (−28.73, −15.63), *P* < .05] in the fixed-effects model analysis.

### 3.5 The subgroup analyses

Subgroup analysis was applied to investigate the effects of different factors on TC. TC decreased significantly in subjects with severe hyperlipidemia, with more prominent effect in those with marginal elevation (200 ≤ TC ≤ 239 mg/dL). The pooled mean net change in TC was [MD = −15.62, 95% CI (−23.65, −7.59), *P* < .05]. Additionally, the results showed that factors of single or multi strains, and small or large dose (<10^9^ cfu and ≥10^9^ cfu, respectively) both presents no significant effect on the mean net change in TC. Compared with probiotics milk (yogurt), the consumption of probiotics capsules significantly reduced TC [MD = −13.79, 95% CI (−18.84, −8.75), *P* < .05]. In addition, the decline in TC levels were greater in the long period intervention groups (*t* ≥ 8 weeks) than in the short period intervention groups (*t* < 8 weeks). The pooled mean net change of long period intervention in TC was [MD = −14.37, 95% CI (−19.44, −9.30), *P* < .05] (Table 2).

**Table 2:**
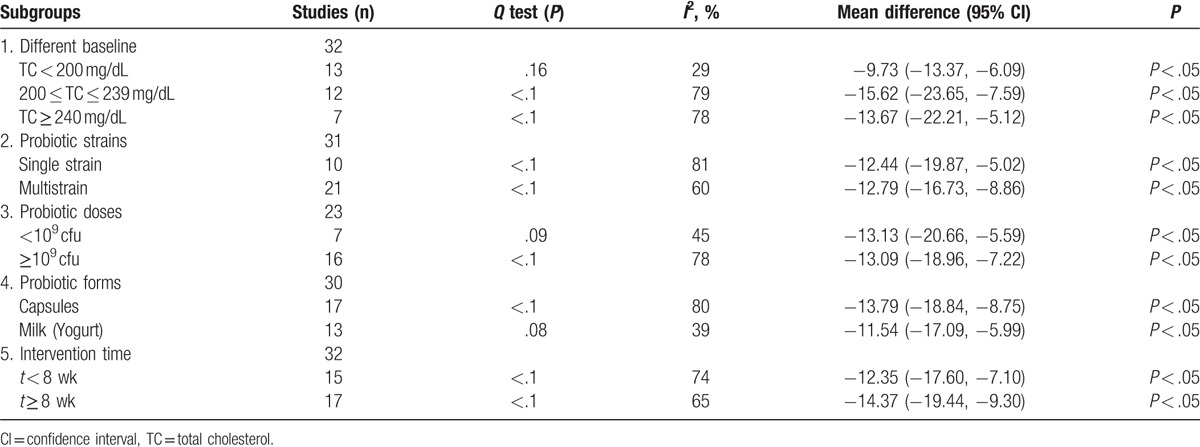
The subgroup analyses of the effect of probiotics on total cholesterol.

### 3.6 Meta-regression

Due to the large heterogeneity of the meta-analysis, a single factor meta-regression (using REML) was performed to find the possible sources of heterogeneity as shown in Table 3. *P* > .05 in all analysis which indicates that the factors involved may not be the sources of heterogeneity.

**Table 3:**
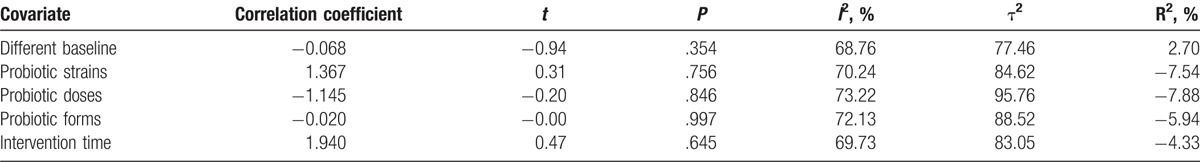
The meta-regression of 5 covariates.

### 3.7 Publication bias

**Figure 6:**
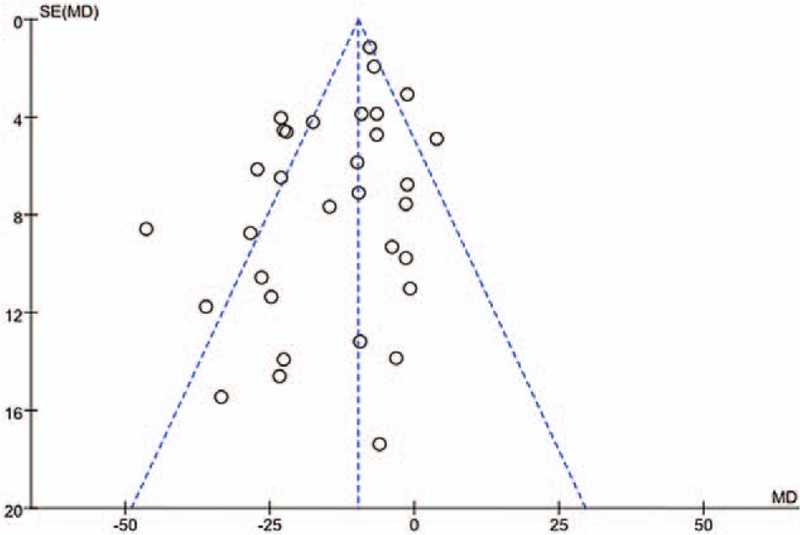
Publication bias Funnel plots were used to determine whether publication bias exists for all of the included studies. The results showed that publication bias might exist in the included trials.

Funnel plots were used to determine whether publication bias exists for all of the included studies. The results can be obtained from the funnel plot as shown in Fig. 6. It is asymmetric, which indicates that publication bias possibly exist in the included trials. Then we calculated the fail-safe number. The result of Nfs0.05 calculation is 1897, that is to say, the need for 1897 negative study can overturn the existing conclusions. This indicates that the publication bias is small and the result is stable.

## 4 Discussion

This meta-analysis of the 32 RCTs concluded that the probiotics group significantly reduced serum TC compared to the control group, which is consistent to previous meta-analysis. It provides an updated and detailed report of the effects of probiotics on serum TC. In the previous meta-analysis conducted by Cho and Kim^[14]^ which include 30 RCTs, it reported the hypocholesterolemic effects of probiotics (7.8 mg/dL). Another report by Shimizu et al^[16]^ showed a mean net change in TC (6.57 mg/dL) using 33 RCTs. Jing et al^[12]^ also demonstrated its lipid lowering effect of the TC with net change of 10.44 mg/dL, using 10 studies. Agerholm-Larsen et al^[11]^ revealed that the probiotics products (Gaio, The Danish Dairy Corporation MD Foods A/S, Aarhus, Denmark) changed TC by 8.51 mg/dL, analyzing 6 studies. While in the study of Sharma et al,^[13]^ the pooled mean net change for TC is 8.40 mg/dL (from 12 trials). Finally, study of Guo et al^[15]^ reported a net change of 6.40 mg/dL in TC (from 13 trials). The results obtained by our meta-analysis presented a pooled mean net change of 13.27 mg/dL in TC upon probiotics taken. Our result shows a more prominent effect than previous studies, which might due to the multiple facts. In our meta-analysis, involving a relatively long intervention time compared to the 6 studies above; wider range of research were included compared to Agerholm-Larsen et al's,^[11]^ Sun's,^[12]^ Sharma et al's,^[13]^ and Guo et al's studies^[15]^; more probiotic types were included compared to Agerholm-Larsen et al's^[11]^ and Sharma et al's studies.^[13]^ These factors may have contributed to the difference in outcomes. Besides, other factors that were not found may also affect the outcome.

This study also analyzed the effect of specific probiotics strains separately. *L acidophilus* and *B lactis* are the most commonly used probiotics. Some studies reported the use of these probiotics. For instance, Shimizu et al^[16]^ reported that the *L acidophilus* strain has greater ability to lower TC. Furthermore, in vitro studies showed that *L acidophilus* and *B lactis* can reduce cholesterol absorption.^[6–8]^ Our study demonstrated similar results. Therefore, *L acidophilus* and *B lactis* can downregulate serum TC level. Additionally, the VSL#3 group and the *L plantarum* group were both shown to be able to reduce TC effectively. Although above pooled results existed heterogeneous, it was resolved by subgroup analysis. *L plantarum* was reported to be able to survive in the environment of acid or bile and colonize easily in human intestine.^[42]^ This information is useful for the future studies involving the TC reducing effect of *L plantarum*. It is worth noting that, the accuracy of the results needs to be further confirmed because of the limited number of articles that were included.

A large heterogeneity among the included studies was detected in this meta-analysis, so we performed subgroup analyses to explore possible reasons for it and found that TC lowering effect of probiotics differs under different conditions.^[43]^ The difference of baseline TC may also have a significant impact on the results. Therefore, subgroup analysis was performed according to the classification standard (NECP) of their baseline TC level. It disclosed that higher TC level (200 ≤ TC ≤ 239 mg/dL and TC ≥ 240 mg/dL) associated with a stronger improvement in TC compared to the lower group (TC < 200 mg/dL). This is consistent with the conclusion of Cho et al.^[12,14,31]^ It might be that the human body is in a pathological state when TC > 200 mg/dL, which could be more sensitive to probiotics. Furthermore, our analysis also included a subgroup with margins TC (200 ≤ TC ≤ 239 mg/dL), which showed a strongest improvement in TC compared to higher group (TC ≥ 240 mg/dL). It is possible that this group is more sensitive to probiotics. Besides, due to the limited quantity of including studies involving groups with high TC, this result might be affected, and the contrast results of the 2 groups remains to be further confirmed by large sample experiment.

What is more, this study detected that different group with single or multiple strains and low dose or high dose did not showed significant difference on TC lowering. This indicated that the strain and dosage of the probiotics did not influence the TC lowering effect. This could be due to the limited quantity of including studies. Besides, the unresolved heterogeneity is due to other factors, which might interfere with the results as well. Therefore, the influence of strain and dosage of the probiotics on TC needs to be further verified using more high-quality and large-sample studies.

The subgroup analyses in our study showed that probiotics in capsules presented with a more significant TC lowering effect than probiotics milk (yogurt). This result is contrary to previous studies.^[12,16]^ For example, Shimizu et al's study,^[16]^ which included 11 articles (in total: 8 capsules subgroup and 4 yogurts subgroup). As well as Sun's study, which only analyzed 10 articles with low statistical efficiency (in total: 3 capsules subgroup and 10 yogurts subgroup).^[12]^ By contrast, our meta-analysis included 30 RCTs (in total: 17 capsules subgroup and 13 yogurts subgroup), with 1875 participants. Moreover, comparing with probiotics yogurt, capsules which has a clear components and the activity of the strains would be not easily influenced by other factors. Therefore, probiotics capsules can reduce TC more significantly than yogurt.

The results of our meta-analysis showed that the long-term probiotics intervention could significantly reduce the level of TC, which was in line with some previous studies.^[9,12,14,16]^ However, other studies suggested that the duration of probiotics consumption had no significant effect on the TC.^[11]^ The difference may be caused by these meta-analysis only including studies with short intervention time (4–8 weeks) but our analysis has a wider range of intervention time (1–24 weeks). Therefore, this information is possibly beneficial in the prevention and treatment of cardiovascular disease in the near future.

But our meta-analysis has several limitations. Heterogeneity still remained, although the combined results were analyzed by meta-regression, sensitivity and subgroup analysis. In addition, there are some deficiencies in the quality of literature, which are likely to impact the final results. Therefore, we recommend that more large sample-sized, randomized multicentric studies are conducted to further our understanding of this interesting and potential beneficial of nonpharmacological alternative for the management of cardiovascular risk in patients.

## 5 Conclusions

The probiotics can significantly reduce serum TC. At the same time, patients with higher TC levels, using probiotics capsules and extending intervention time may be more beneficial to the outcome.
